# Long-term probucol therapy continues to suppress markers of neurovascular inflammation in a dietary induced model of cerebral capillary dysfunction

**DOI:** 10.1186/1476-511X-13-91

**Published:** 2014-06-03

**Authors:** Ryusuke Takechi, Menuka M Pallebage-Gamarallage, Virginie Lam, Corey Giles, John CL Mamo

**Affiliations:** 1 School of Public Health, and CHIRI Biosciences Research Precinct, Faculty of Health Sciences, Curtin University, Bentley, GPO Box U1987, Perth 6845, WA, Australia

**Keywords:** Brain capillaries, Amyloid-β, Apolipoprotein B, Blood–brain barrier, Enterocytes, Neuroinflammation, Probucol, Saturated fatty acids

## Abstract

**Background:**

Probucol has been shown to prevent cerebral capillary disturbances characterized by blood-to-brain extravasation of plasma derived proteins and neurovascular inflammation in mice maintained on western-styled diets for 12 weeks. However the effect of probucol on capillary integrity in aging models with capillary dysfunction is not known.

**Methods:**

Wild-type C57BL6 mice were randomized to a low-fat (LF); saturated-fat (SFA); or SFA + Probucol diet for up to12 months of intervention.

**Results:**

Mice fed the LF diet had substantially greater parenchymal abundance of plasma derived IgG and apo B lipoproteins at 12 months, compared to LF mice at 3 months of intervention. Markers of neurovascular inflammation were also greater at 12 months in LF fed mice compared to LF mice at 3 months. The SFA diet exacerbated the aging induced parenchymal abundance of IgG and of apo B lipoproteins and neurovascular inflammation at 12 months. The SFA effects were associated with increased production of intestinal lipoprotein amyloid-β (Aβ). The co-provision of probucol with the SFA completely abolished heightened inflammation at 12 months. Probucol attenuated SFA-induced capillary permeability but had only a modest inhibitory effect on parenchymal retention of apoB lipoproteins. The improvements in markers of inflammation and capillary integrity because of probucol correlated with enterocytic genesis of chylomicron Aβ.

**Conclusion:**

In this long-term feeding study, probucol profoundly suppressed dietary SFA induced disturbances in capillary integrity but had a more modest effect on age-associated changes.

## Background

Probucol is an older generation cholesterol-lowering agent once commonly prescribed to reduce cardiovascular disease risk before the advent of HMGCoA-reductase inhibitors (statins). However, some studies suggest that probucol may also have neurovascular benefits. Probucol was found to prevent cognitive and hippocampal synaptic impairments induced by amyloid-beta (Aβ) peptides in mice [1] and in a pilot clinical study in subjects with early-mid Alzheimer’s disease (AD), probucol was reported to stabilize cognitive decline compared to the placebo treated group [2]. The mechanisms for the purported positive effects of probucol in AD and in models of AD are unknown but are suggested to include decreased formation of toxic Aβ oligomers as a consequence of increased Aβ chaperoning [3], or direct positive effects of the agent on neurovascular integrity and function [4].

In wild type mice, the short-term (3 months) ingestion of a diet either supplemented with cholesterol (1% w/w) or modestly enriched in saturated fatty acids (SFA, 20% w/w) induced a substantial disruption of blood–brain barrier (BBB) integrity, resulted in accumulation of plasma-derived proteins within brain parenchyme [4-6]. The SFA diet had a more profound detrimental effect on capillary permeability compared to the cholesterol-supplemented diet, which occurred concomitant with increased cerebral abundance of Aβ that was associated with apoB lipoproteins. Given that probucol was found to markedly suppress enterocytic production of lipoprotein associated Aβ following 3 months of SFA-feeding [7], decreased vascular post-prandial exposure to Aβ associated with apoB lipoproteins may have been one mechanism by which probucol maintained cerebral capillary function.

Recent studies suggest that in wild-type mice maintained on a low-fat (LF, cholesterol and SFA-free) diet, capillary permeability will progressively increase 4–5 fold over a 12-month period compared to mice at 3 months of age [8]. However, interactive effects of aging with dietary-fats were reported and include accelerated BBB permeability as a consequence of the ingestion of SFA. The study also demonstrated that exaggerated blood-to-brain kinetics of plasma proteins as a consequence of aging and fat-feeding results in heightened neurovascular inflammation. Given these findings and in the context of further considering the potential therapeutic value of probucol in attenuating neurovascular inflammatory disorders associated with ageing and western-styled diets, longer-term studies are needed. This study considered the efficacy of probucol in C57BL/6 mice maintained on LF fed or SFA-fed diet for 12 months.

## Results

**Figure 1:**
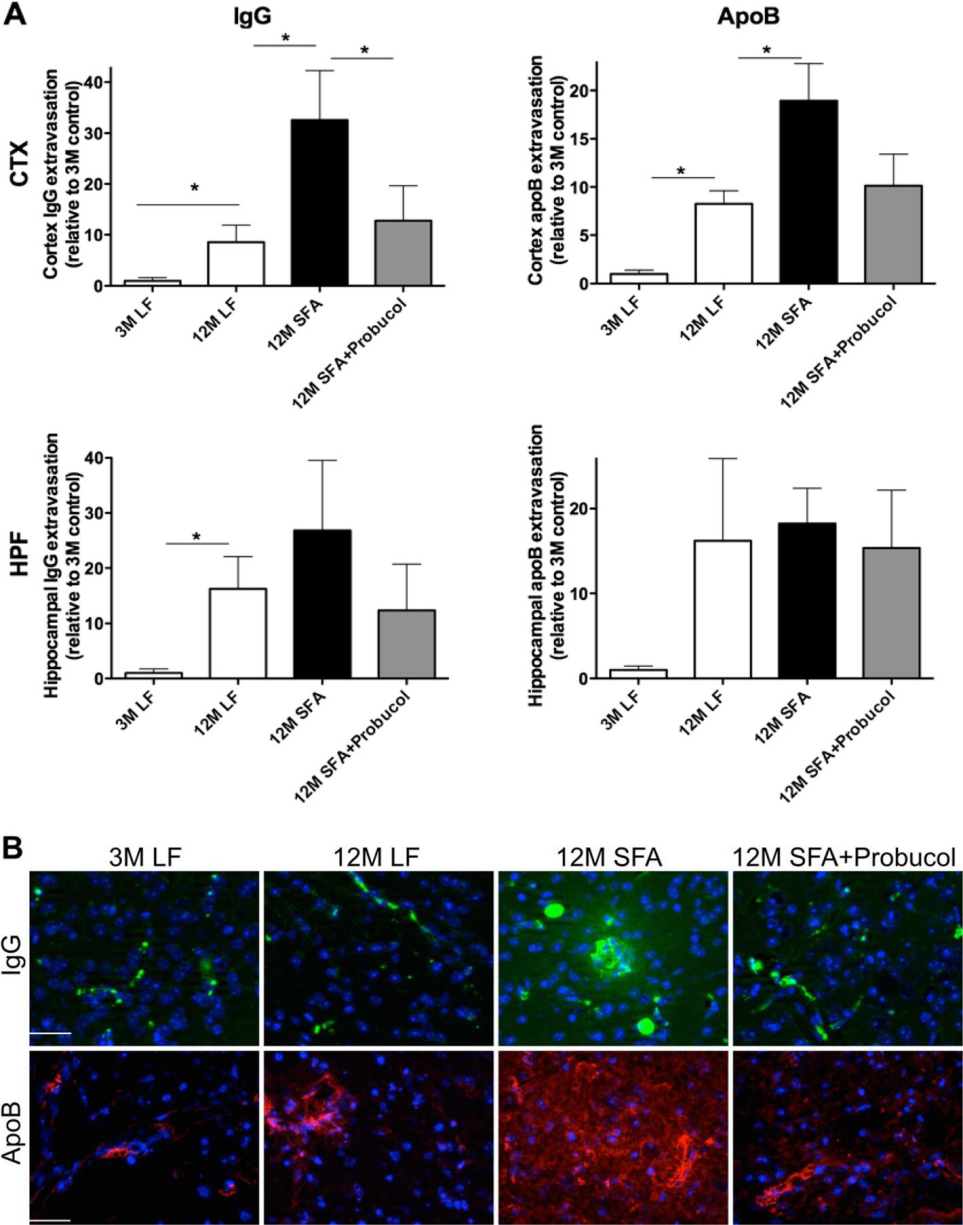
**Semi-quantitative 3-D immunomicroscopy for the assessment of blood–brain barrier integrity.** The integrity of blood–brain barrier (BBB) in mice maintained on low-fat (LF) control chow or diet enriched in saturated fatty acids (SFA) with or without probucol for 3 months (3 M) and 12 months (12 M) was assessed by the cerebral extravasation of plasma proteins, IgG and apolipoprotein (apo) B. **(A)** 3-D quantitative immunomicroscopy graphs show the mean voxel intensity of IgG/apoB that are in the parenchyme of cortex (CTX) and hippocampal formation (HPF). The * shows the statistical significance assessed by one-way ANOVA with Tukey’s post hoc test (*p <* 0.05, n = 8). **(B)** Representative images of capillary vessel integrity are provided. IgG is shown in green, apolipoprotein (apo) B is in red, and nuclei are in blue. The scale bar indicates 100 μm.

**Figure 2:**
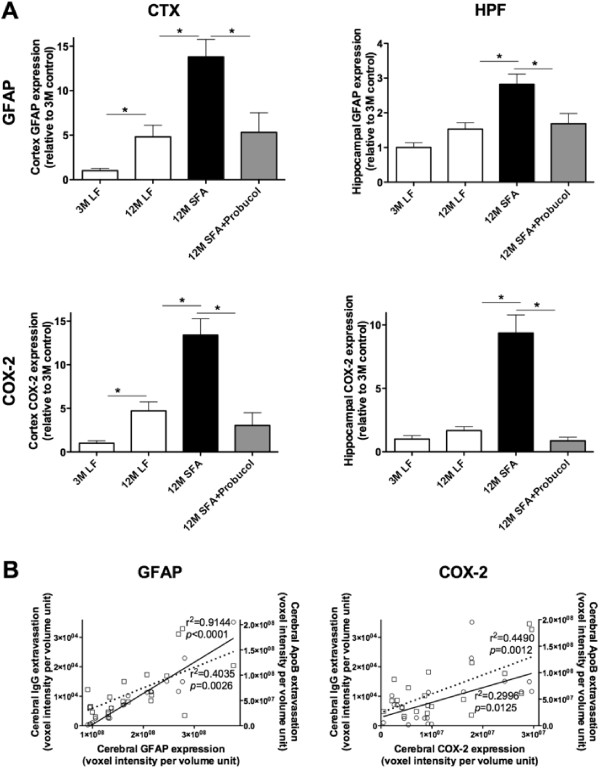
**Semi-quantitative 3-D immunomicroscopy for cerebrovascular and neuronal inflammation.** Cerebrovascular and neuronal inflammation was assessed by 3-D immuno quantitative analysis of the cerebral parenchymal and vascular expressions of GFAP and COX-2 in cortex (CTX) and hippocampus (HPF) of mice fed with low-fat (LF) or saturated fat (SFA) diet with/without probucol for 3 and 12 months. **(A)** Mean voxel intensity of parenchymal GFAP and COX-2 in CTX and HPF is shown. * indicates the statistical significance assessed with one-way ANOVA followed by Tukey’s post hoc test (*p <* 0.05, n = 8). **(B)** Pearson’s correlation coefficient between the GFAP or COX-2 expression and cerebral IgG/apoB extravasation in each animal was also performed (n = 24).

**Figure 3:**
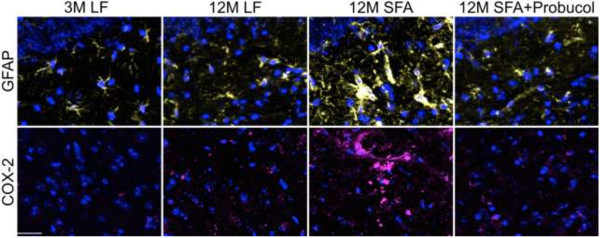
**Immunofluorescent micrographs of cerebral GFAP and COX-2 expression.** Representative images of neuroinflammatory GFAP and COX-2 in mice maintained on low-fat (LF) control chow or diets enriched in saturated fats (SFA) with/without probucol for 3 months (3 M) and 12 months (12 M) are shown. GFAP is shown in pale yellow, COX-2 is in magenta, and nuclei are in blue. The scale bar indicates 100 μm.

The body weights, plasma lipids and non-esterified fatty acids for the treatment groups are provided in Table 1. Body weights were comparable at 12 months of intervention irrespective of dietary regimen, although weight gain was transiently greater in mice fed the SFA enriched diets between 3–12 months of feeding. The SFA diet was well tolerated and mice were normo-lipidemic throughout the duration of dietary intervention. Plasma non-esterified fatty acid (NEFA) were increased in SFA fed mice at 3 months, but were comparable to LF fed controls at 12 months of feeding (Table 1). Whilst the SFA fed diet had no significant impact on plasma cholesterol, co-provision of probucol nonetheless lowered plasma cholesterol by approximately 80%.In this study, brain parenchymal abundance of IgG was used as a measure of non-specific capillary permeability. ApoB lipoproteins are exceedingly large macromolecules that become enriched with Aβ as a consequence of SFA feeding. Figure 1A compares the parenchymal abundance of IgG and apoB lipoproteins in mice maintained on an SFA enriched diet 12 months relative to LF fed control mice at 3 months. The synergistic effects of probucol with SFA feeding on parenchymal IgG and apoB are also illustrated in Figure 1A. Representative images from each treatment group are provided as Figure 1B. We firstly confirm that at 12 months of intervention, IgG and apoB extravasation in LF fed mice was significantly greater compared to 3 months, indicative of an age-specific effect. The synergistic effects of SFA with age remained evident for IgG and apoB within the CTX (Figure 1). We now show that at 12 months of SFA feeding, probucol continued to suppress parenchymal accumulation of IgG, however there was only a modest inhibitory effect on retention of apoB lipoproteins within brain parenchyme.The effects of probucol on established markers of neurovascular inflammation (glial fibrillary acidic protein (GFAP) and cyclooxygenase-2 (COX-2)) in mice fed the LF or SFA-enriched diet are shown in Figures 2A and 3. Consistent with our previous finding, GFAP within the cortex were increased in LF fed mice at 12 months of age compared to 3 months of age. We now also report a significant increase of COX-2 in the CTX by aging. The SFA enriched diet markedly increased GFAP and COX-2 expression within both brain regions of interest at 12 months of feeding. Probucol completely abolished the SFA induction of GFAP and COX-2. Potential associations between inflammation (GFAP and COX-2) and measures of capillary permeability and brain parenchymal apoB retention are indicated in Figure 2B. The correlation coefficients for GFAP versus cerebral IgG and apoB extravasation were significant. Similarly, COX-2 expression was also positively associated with parenchymal IgG and apoB.

**Table 1:** Weights, plasma lipids, and non-esterified fatty acids

	**3 months**			**12 months**		
	**LF**	**SFA**	**SFA + Probucol**	**LF**	**SFA**	**SFA + Probucol**
Weight (g)	22.2 ± 0.33	22.7 ± 0.33	22.7 ± 0.33	30.8 ± 1.5	36.4 ± 1.0	32.8 ± 2.5
Weight gain (g)	-	-	-	14.3 ± 1.50	21.3 ± 1.02*	13.26 ± 2.53
Cholesterol (mmol/L)	3.18 ± 0.26	3.13 ± 0.22	0.58 ± 0.074**	3.25 ± 0.29	2.72 ± 0.12	0.58 ± 0.068**
Triglyceride (mmol/L)	0.35 ± 0.023	0.32 ± 0.038	0.44 ± 0.034	0.51 ± 0.082	0.45 ± 0.11	0.56 ± 0.13
NEFA (mEq/L)	0.49 ± 0.064	0.70 ± 0.041**	0.34 ± 0.041	0.47 ± 0.026	0.40 ± 0.021	0.44 ± 0.051

LF = Low fat, SFA = Saturated fatty acid, NEFA = Non-esterified fatty acid.

Data are shown as mean ± SEM. Statistical significance analyzed with one-way ANOVA followed by Tukey’s post hoc test (2 tailed, n = 8) is indicated at *p <* 0.05 (*) or at *p <* 0.01 (**).

**Figure 4:**
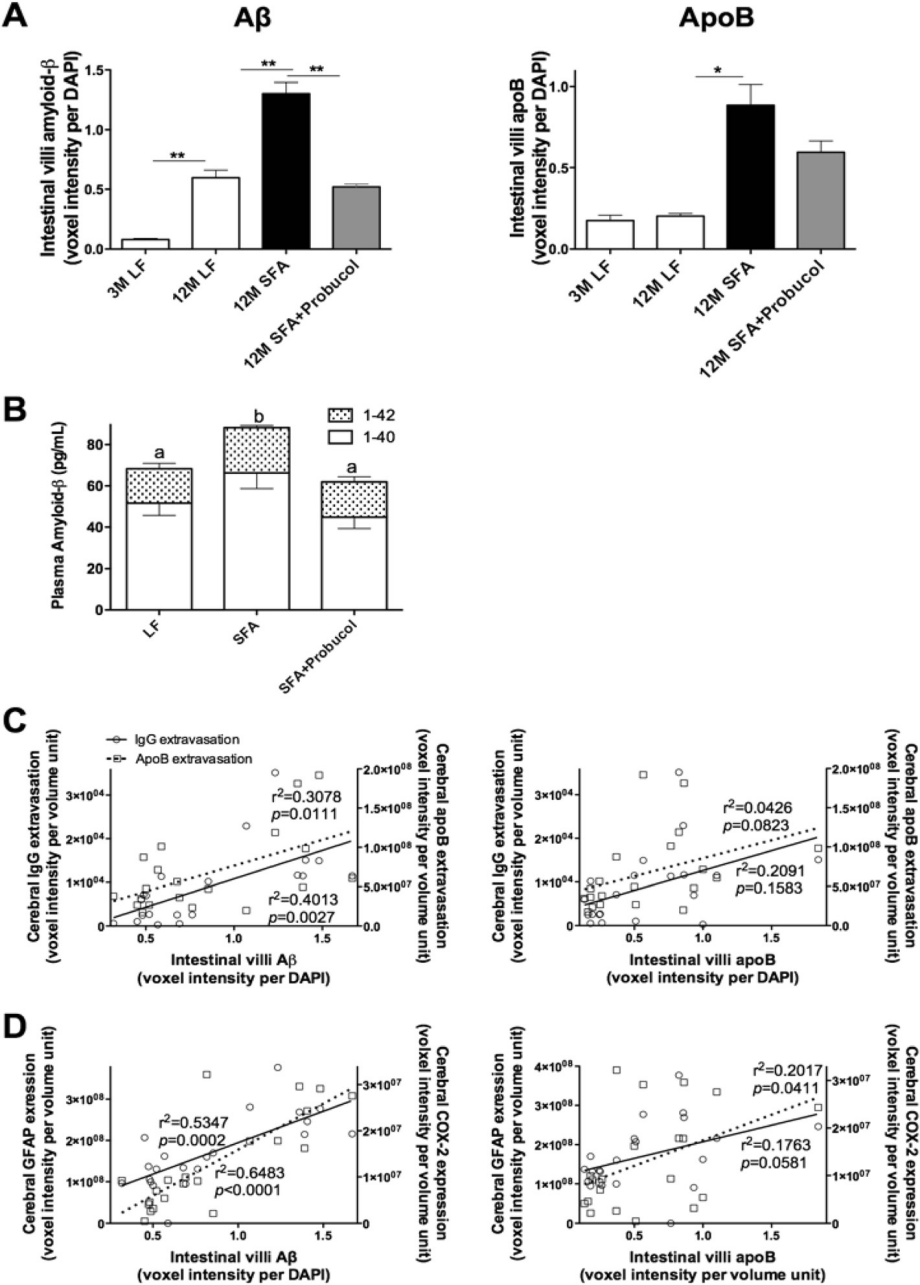
**Semi-quantitative 3-D immunomicroscopy for small intestinal production and secretion of amyloid-β and apolipoprotein B, and plasma amyloid-β.** The production within the enterocytes and secretion into the lacteal of amyloid-β (Aβ) and apolipoprotein (apo) B were determined with 3-D quantitative immunomicroscopy in the small intestine of mice maintained on low-fat (LF) control chow or diets enriched in saturated fats (SFA) with/without probucol for 3 months (3 M) and 12 months (12 M). **(A)** The mean voxel intensity of small intestinal villi Aβ or apoB are expressed per DAPI voxel intensity. One-way ANOVA followed by Tukey’s post hoc test was performed and the significance is indicated with * (p < 0.05, n = 8). **(B)** The plasma concentration of Aβ was measured with ELISA. One-way ANOVA followed by Tukey’s post hoc test was performed to compare the difference of total plasma Aβ, and the significance is indicated with alphabetical letters (p < 0.05, n = 8). **(C)** Correlation coefficient between intestinal Aβ or apoB with cerebral IgG/apoB extravasation are also shown. Correlation coefficient was determined with Pearson’s analysis (n = 24). **(D)** Correlation coefficient between intestinal Aβ or apoB with neuroinflammatory GFAP/COX-2 are also shown.

**Figure 5:**
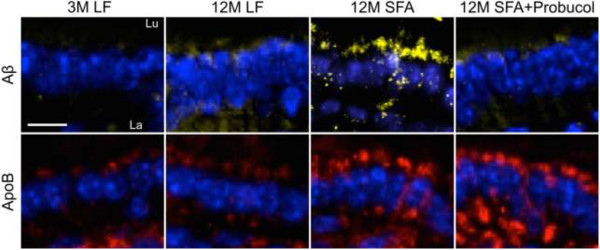
**Immunofluorescent micrographs of small intestinal Amyloid-β and apolipoprotein B.** Representative immunofluorescent micrographs of small intestinal amyloid-β (Aβ) and apolipoprotein (apo) B are shown in mice maintained on low-fat (LF) control chow or diets enriched in saturated fats (SFA) with/without probucol for 3 months (3 M) and 12 months (12 M). Aβ and apoB are shown in yellow and red respectively, and the nuclei are shown in blue. “Lu” and “La” indicate intestinal lumen and villi lacteal respectively, and the cell lining between Lu and La are the absorptive enterocytes. The scale bar indicates 10 μm.

To explore if the persistent parenchymal abundance of apoB at 12 months of feeding was associated with the biogenesis of enterocytic production of lipoprotein AB and to explore whether this was one mechanism by which probucol regulated parenchymal retention of plasma derived proteins and inflammatory sequelae, the intestinal production of lipoprotein Aβ was determined by measuring the abundance of enterocytic Aβ and apoB. Mice fed the LF diet at 12 months of intervention had a remarkable 6-fold elevation in enterocytic Aβ compared to mice at 3 months of intervention. The substantial increase in enterocytic Aβ however, occurred without any apparent change in lipoprotein abundance per se (indicated by enterocytic apoB) (Figures 4A and 5). Provision of a diet enriched in SFA doubled the age-induced effect on enterocytic Aβ. However, in addition the SFA diet also strongly stimulated chylomicron biosynthesis. The co-provision of probucol with SFA was found to substantially attenuate the SFA-induced enterocytic abundance of Aβ by more than 40% (Figures 4A and 3). Probucol showed more modest suppressive effects on enterocytic chylomicron apoB abundance. Consistent with the findings in enterocytes, the plasma concentration of Aβ_1–40_ and _1–42_ were significantly elevated with 12 months SFA ingestion, but suppressed by the co-provision of probucol (Figure 4B).Figure 4C considers capillary permeability in the context of a possible association with postprandial chylomicron Aβ metabolism. The findings indicate a reasonably strong positive correlation between enterocytic Aβ and cerebral capillary permeability. However, the correlation analysis did not support an association between parenchymal abundance of apoB lipoproteins with chylomicron particle concentration per se (ie. enterocytic apo B) (Figure 4C). Furthermore, there was a strong correlation of GFAP and COX2 with enterocytic chylomicron Aβ. There was also an association of inflammation with chylomicron abundance (apoB), however the correlation coefficient was markedly weaker (Figure 4D).

## Discussion

This study investigated in a murine model the efficacy of probucol to suppress cerebral capillary dysfunction and heightened neurovascular inflammation that occurs with aging and long-term ingestion of SFA enriched diets.

Previous studies have equivocally demonstrated the presence of plasma-derived proteins within brain parenchyme in mice maintained on SFA enriched diets for 3 to 12 months [4,5,8-10]. Diets enriched in SFA also promote secretion from epithelial cells of the small intestine of Aβ associated with chylomicrons [7,11,12] and these findings previously led us to propose that exaggerated exposure to SFA and/or chylomicron-Aβ compromise capillary permeability. Whatever the mechanism, plasma derived apoB lipoprotein-Aβ extravasation to brain parenchyme and exaggerated retention upon extracellular matrices has been demonstrated in mice fed an SFA-enriched diet for 6 months [5]. In more recent studies, synergistic effects of aging and western styled diets on cerebral capillary dysfunction were demonstrated [8]. Notably however, marked capillary dysfunction was also shown in aged (12 month) mice maintained on a LF diet that was free of saturated fats and cholesterol.

Several lines of evidence suggest that probucol may be an agent of therapeutic value for individuals with AD [2]. In addition to positive capillary function regulatory effects in mice maintained for relatively short periods of time on SFA/cholesterol diets, probucol has other potentially useful metabolic effects including synaptic potentiation [1]; inhibition of Aβ biogenesis [7]; promotion of Aβ-chaperoning (reduction in formation of toxic oligomers) [3]; vasodilatory effects [13]; and anti-oxidant/-inflammatory properties [4,13]. However, further consideration of probucol in a therapeutic context for prevention of non-familial AD would be strengthened by longitudinal *in vivo* studies in an aging context.

This study used an established model of BBB dysfunction induced by aging and exacerbated by the provision of dietary SFA to explore the effects of probucol on cerebral capillary function. The dietary intervention was physiologically relevant with approximately 40% of energy derived as fats. The SFA diet was well tolerated and mice randomized to this treatment were found to be normolipidemic and had similar body weight to LF fed controls at the conclusion of treatment. Probucol predictably lowered plasma cholesterol but this was not associated with either parenchymal extravasation of plasma proteins, or measures of neurovascular inflammation and so was not considered further.

In this study, we firstly confirm that parenchymal extravasation of plasma derived IgG is increased as a consequence of aging and exacerbated by SFA feeding consistent with our previous study [8]. We extend those findings and now show that parenchymal retention of apoB lipoproteins is markedly increased in mice maintained for 12 months on an otherwise healthy LF diet. A synergistic effect of aging with SFA resulted essentially in a doubling of the age-induced effect. The co-provision of probucol with SFA was found to completely abolish the long-term SFA induced effect, a phenomenon previously reported in mice maintained on an SFA diet for just 3 months [4]. However, probucol had no marked effect on the age-associated increased abundance of plasma-derived proteins within brain parenchyme, only showing parenchymal IgG and apoB reduction to the 12 months LF level but not to the 3 months LF mice without the ageing effect. Some regional differences in effectiveness were also noted. Probucol effectively abolished the SFA induced accumulation of IgG and apoB within the CTX following 12 months of intervention. However, within the HPF probucol had only modest but not significant effect on apoB retention at 12 months of intervention.

GFAP and COX-2 are widely used measures of neurovascular inflammation. Both measures were increased principally within the cortex in mice maintained on a LF diet for 12 months compared to those following 3 months of intervention. However, GFAP and COX-2 were markedly elevated as a consequence of SFA in both CTX and HPF. The co-provision of probucol had a remarkable suppressive effect on these measures of neurovascular inflammation essentially completely abolishing the SFA-induced effect that persisted for the duration of intervention. Collectively, probucol appears to protect BBB integrity through the suppression of neurovascular inflammation in aged mice maintained on an SFA diet.

Another possible mechanism by which probucol prevents the long-term high SFA induced BBB dysfunction may attribute to the reduced BBB exposure to circulating Aβ that are associated with apoB lipoproteins. Indirect evidence comes from studies in amyloid transgenic mice, where it was reported that onset and progression of amyloidosis was positively associated with the secretion into blood of Aβ that was associated with nascent chylomicron [14]. The SFA diet used in this study was previously demonstrated to increase the enterocytic association of Aβ with nascent chylomicrons [11,12]. In other studies; probucol was shown to strongly suppress the chylomicron-Aβ in mice fed an SFA enriched diet for 3 months [7]. Hence the proposition that postprandial-Aβ is associated with age-induced capillary dysfunction is worthy of consideration.

Enterocytic apoB (an obligatory structural element of chylomicrons) is a useful surrogate marker of chylomicron biosynthetic rates [15]. The abundance of Aβ that is colocalized with nascent chylomicrons is therefore a direct measure of protein abundance per lipoprotein particle assembled.

This study reports that mice maintained on a LF diet for 12 months had substantially elevated Aβ relative to nascent chylomicrons within the absorptive epithelial cells of the small intestine compared to 3 months LF control mice. However, enterocytic apoB production remained comparable. Long-term ingestion of SFA enriched diets doubled the enterocytic postprandial Aβ production concomitant with increased enterocytic apoB lipoprotein, suggesting increased release of lipoproteins and Aβ into the circulation. However, the enterocytic production of Aβ was completely abolished by the co-provision of probucol with the SFA diet. In mice, it is not feasible and perhaps not relevant to measure postprandial chylomicron-Aβ in plasma because of the limited sampling volume; separation of plasma lipoprotein classes; the effects of anesthesia and mimicking the ‘chronic’ (12 month) dietary intervention in the context of a singular acute fat challenge. Nonetheless, our data showed a significant increase in the plasma Aβ by the long-term ingestion of SFA enriched diet. The study also provided evidence of a quite strong positive association between chylomicron-Aβ, cerebral-capillary permeability and parenchymal apoB lipoprotein abundance. Consistent with the notion that chylomicron-Aβ per se, rather than chylomicron concentration is more relevant to capillary integrity, there was a relatively poor positive association of permeability with enterocytic apoB. Furthermore, probucol had a substantial effect on enterocytic Aβ, but a weaker and non-significant effect on apoB abundance. These data collectively suggest that probucol may protect the BBB integrity through reduced BBB exposure to postprandial lipoprotein-Aβ by attenuating the enterocytic production of postprandial Aβ.

This is the first long-term intervention study showing potential beneficial effects of probucol on cerebral capillary integrity in the context of reducing risk for Alzheimer’s disease. This study demonstrates persistent suppression by probucol of neurovascular inflammation and differential effects of capillary permeability in aged mice that had been maintained on an SFA-enriched diet. The beneficial effects of probucol on SFA-induced capillary dysfunction may in part be a consequence of suppression of postprandial lipoprotein-Aβ secretion and decreased vascular exposure, and of its anti-oxidative/-inflammatory properties.

## Materials and methods

### Animals and dietary/drug interventions

Wild-type female C57BL/6 J mice (6 weeks old) were purchased from Animal Resources Centre (WA, Australia). Groups of 16 mice were randomized to treatment groups of LF control, high SFA, or high SFA supplemented with probucol. The LF diet was standard rodent chow (AIN93M) containing 4% polyunsaturated fats without cholesterol or SFA. The SFA diet was semi-synthetic chow contained 40% total energy as saturated fats derived from cocoa butter (5% w/w palmitic 16:0, 7% stearic 18:0) as described previously [4] and shown in Table 2. Probucol was supplemented to the SFA diet at a concentration of 1% (w/w), determined according to previous studies [4,7,16,17]. Diets were prepared and supplied by Specialty Feeds (WA, Australia). Probucol was kindly provided by Sanofi-Aventis (France). The mice were housed in individually ventilated cages with controlled temperature (22˚C), air pressure, and 12 h dark/light cycle, and had ad libitum access to the food and water.

**Table 2:** Dietary composition of AIN93M and SFA diets

	**AIN93M**	**SFA**
Ingredients (g/kg)		
Casein	140	140
DL methionine	1.8	1.8
Sucrose	100	100
Wheat starch	472	308
Dextrinised starch	155	155
Cellulose	50	50
Canola oil	40	0
Cocoa butter	0	204
Calcium carbonate	13.1	13.1
Sodium chloride	2.6	2.6
Potassium citrate	1.0	1.0
Potassium dihydrogen phosphate	8.8	8.8
Potassium sulphate	1.6	1.6
AIN93G trace minerals	1.4	1.4
Choline chloride (65%)	2.5	2.5
AIN93G vitamins	10	10
Fat composition (w/w)		
Total fat	4%	
Saturated fatty acids C12:0 and less	na	0.10%
Myristic acid 14:0	trace	0.05%
Pentadecanoic acid 15:0	na	0.01%
Palmitic acid 16:0	0.2%	5.16%
Magaric acid 17:0	na	0.05%
Stearic acid 18:0	0.1%	7.31%
Arachidic acid 20:0	na	0.24%
Behenic acid 22:0	na	0.04%
Tetracosanoic acid 24:0	na	0.03%
Palmitoleic acid 16:1	trace	0.05%
Heptadecenoic acid 17:1	na	0.01%
Oleic acid 18:1 n9	2.4%	6.62%
Gadoleic acid 20:1	trace	0.01%
Linoleic acid 18:2 n6	0.8%	0.67%
a Linolenic acid 18:3 n3	0.4%	0.05%
g Linolenic acid 18:3 n6	na	not detected
Arachadonic acid 20:4 n6	trace	not detected
EPA 20:5 n3	trace	not detected
DPA 22:5 n3	na	not detected
DHA 22:6 n3	trace	not detected

All procedures used in this study were approved by National Health and Medical Research Council accredited Animal Ethics Committee (Curtin University approval no. R34/08).

### Sample collection and preparation

Samples were collected and prepared as described previously [5,11]. Briefly, at 3 and 12 months following dietary/drug intervention, 8 mice from each intervention group were euthanized under complete anesthesia with pentobarbitone (45 mg/kg). Plasma sample was collected from cardiac puncture. The brain tissues were collected as described previously [5,6,8]. Briefly the brain was carefully removed and washed in ice-chilled PBS as previously described. The tissues were fixed in 4% paraformaldehyde immediately to minimize the post-mortem artifact for 24 h, then cryoprotected in 20% sucrose for 72 h at 4˚C. The tissues were then frozen in dry ice/isopentane and stored at -80˚C. In all mice, the first centimeter of small intestine was also isolated and washed in ice-chilled PBS and fixed in 4% paraformaldehyde for 24 h. The small intestinal tissues were then processed in a tissue processor (TP1020, Leica Australia) and embedded in paraffin wax.

### Assessment of blood–brain barrier integrity

The integrity of BBB was assessed by the cerebral extravasation of plasma-derived proteins, IgG (Mw 155 kDa) and apoB lipoproteins (molecular weight >2 × 10^7^ kDa) as described previously [4-6,8,18,19]. Briefly, 20 μm cryosections were prepared from the right hemisphere in the approximate stereotaxic areas of 1.7 mm interaual and 2.1 mm Bregma of each mouse brain. After a blocking with 10% goat serum in PBS for 30 min, the sections were incubated with goat anti-mouse IgG conjugated with Alexa488 (1:50, Invitrogen, US) for 20 h at 4˚C for IgG analysis. For apoB analysis, the sections were incubated with rabbit anti-mouse apoB (1:200, Abcam, UK) for 20 h at 4˚C. Goat anti-rabbit IgG conjugated with Alexa488 (1:200, Invitrogen) was then applied to the sections for 2 h at 22˚C. DAPI staining was used to identify the cerebrovascular endothelial cells. Negative controls were included for all immunofluorescent experiments and included replacement of the primary antibody with buffer, or an irrelevant serum. Fluorescent staining was not observed for any negative control tissue preparations.

At a magnification of 200× (20× Zeiss Plan-Neofluar objective with 10× mRM camera, 430 × 322 μm), a minimum of five images per section were captured from randomly selected areas of the cortex and hippocampal formation respectively in the approximate stereotaxic areas of 1.7 mm interaual and -2.1 mm Bregma. Each 3-D image consisted of 12 Z-stack images and the distance between Z-stack slices was 1.225 μm optimized by Nyquist theory (2× oversampling in axial direction). The optical densitometric sum for the protein of interest was determined in three dimensions (1388 × 1040 pixels 2D planes) utilizing the measurement tool of image analysis software Volocity ver5 (PerkinElmer, UK). Fluorescence within capillary vessels is excluded based on pre-set threshold parameters and thereafter confirmed for each image to ensure proper selection for parenchymal regions of interest. Total optical densitometric for each 3-D image was expressed as voxel intensity per volume unit, and the mean of all images from each region was used for the statistical comparison.

### Measurement of neuroinflammation

The neuronal inflammatory responses following the disruption of BBB were measured with the immunodetection of GFAP and COX-2 as described previously [4,6]. The 20 μm cryosections were blocked with 5% goat serum for 30 min. Either of rabbit anti-mouse GFAP (1:200, Abcam) or rabbit anti-mouse COX-2 (1:200, Abcam) antibody was added onto the sections, and incubated for 20 h at 4˚C. Goat anti-rabbit IgG conjugated with Alexa488 (1:200) was then applied to the sections for 2 h at 22˚C. Semi-quantitative parenchymal abundance of GFAP and COX-2 were determined within the HPF and CTX as described for IgG and apo B.

### Detection of small intestinal production/secretion of Aβ and apoB

The immunofluorescent technique was utilized to detect the production and secretion of Aβ and apoB from the small intestine as described previously [7,20,21]. Briefly, 5 μm thick paraffin embedded small intestinal sections were dewaxed for 1 h in xylene. After rehydration of sections, the heat mediated antigen retrieval was performed in boiling water for 2 min. Following blocking with 20% goat serum in PBS, the sections were incubated with either rabbit anti-mouse Aβ (1:1000, Chemicon, US) or rabbit anti-mouse apoB (1:300, Abcam) for 20 h at 4˚C. For Aβ detection, the sections were incubated with goat anti-rabbit Ig conjugated with biotin (1:2000, DAKO) for 1 h, followed by incubation with avidin-Alexa546 (1:300, Invitrogen) for 1 h. For apoB detection, the sections were incubated with anti-rabbit IgG Alexa488 (1:200, Invitrogen) for 1 h. The nuclei were stained with DAPI. The staining in the enterocytes (production) and within the lacteal (secretion) was observed with Axiovert 200 M. Semi-quantitative abundance of enterocytic Aβ and apo B were determined as described for parenchymal abundance of IgG and apoB.

### Plasma lipids

The concentration of plasma total cholesterol and triglycerides were measured with colorimetric assays as described previously by using commercially available kits (Randox, UK). Briefly, 2 μl of plasma samples or standards were loaded to 96-well microplates. 200 μl of reaction solution was then added and incubated for 5 min at 37˚C. The optical absorbance was read at 550 nm.

Plasma NEFA were measured by a colourimetric assay purchased from Wako according to the instructions provided by the manufacturer. Briefly, 7 μl of plasma samples or standards were loaded to 96-well microplate. 300 μl of Reagent 1 was added and incubated for 3 min at 37˚C, then 150 μl of Reagent 2 was added for 4.5 min at 37˚C. The optical absorbance was read at 550 nm.

### Plasma amyloid-β

Plasma concentrations of mouse Aβ_1–40_ and _1–42_ were measured utilizing ELISA kits (Invitrogen, US) according to the manufacturer’s instruction. One hundred micro-liter of 4-fold diluted plasma or Aβ standards (Aβ_1–40_: 0, 1, 2.5, 5.0, 10.0, 25.0, 50.0, 100 pmol/L; Aβ_1–42_: 0, 0.1, 0.5, 1.0, 2.0, 5.0, 10.0, 20.0 pmol/L) were dispensed into wells and incubated for overnight at 4˚C, then thoroughly washed. The Aβ antibody conjugated with HRP was then added and incubated for 1 h at room temperature. TMB solution was added for 30 min in darkness, then the reaction was terminated by adding the stopping solution. The optical absorbance was measured at 450 nm.

### Statistics

The number of the animals and immunomicrograph samples collected for the quantitative analyses were determined to produce sufficient statistical power to investigate the effects of Dietary/Drug interventions and Duration/Aging that were also based on the published previous studies [5,6,8]. In this study the data was normally distributed, hence one-way ANOVA followed by Tukey’s or Bonferroni’s post hoc tests were conducted to determine the statistical significance at *p <* 0.05 or *p <* 0.01 (2-tailed).

## Competing interests

The authors declare that there is no conflict of interest of any prior publication of any materials presented herein. All authors have seen and support the publication of this manuscript.

## Authors’ contributions

RT carried out the design of project, collection and analyses of samples and data, statistical analysis and drafting of the manuscript. MP-G and VL assisted in the collection of samples, interpretation of data and preparation of manuscript content. CG helped in the statistical analysis of data and critically analyzing the manuscript content. JM conceived the study, helped in the interpretation of data, drafting of the manuscript, acquiring funding and role in general supervision of the research group. All authors read and approved the final manuscript.
